# Behavior-Opportunity Gaps and Cognitive Health: Findings From a Geographically-Linked Longitudinal Study

**DOI:** 10.1093/geroni/igaf122.1675

**Published:** 2025-12-31

**Authors:** Olivia Atherton, Priscilla Whang, Emily Willroth

**Affiliations:** University of California Riverside, Riverside, California, United States; University of California, Riverside, Riverside, California, United States; Washington University in St. Louis, St. Louis, Missouri, United States

## Abstract

Approximately 40% of Alzheimer’s Disease cases in the U.S. are due to modifiable lifestyle factors such as physical inactivity, heavy alcohol use, and smoking. As such, researchers have begun to focus on how to change individuals’ lifestyle behaviors to improve cognitive health and reduce dementia risk. However, sustained engagement in healthier behaviors can be hindered by environmental constraints (or promoted by environmental opportunities) such as living in an area with few sidewalks, parks, and gyms, or more liquor stores and bars. We refer to this potential discrepancy between health behavior engagement and environmental opportunities as a behavior-opportunity gap. Using data from a large longitudinal study of U.S. older adults (Health and Retirement Study) with geographical linkages, we aimed to characterize behavior-opportunity gaps and their associations with cognitive health in two domains: physical activity and substance use (N = 20,289 participants nested within 5,874 census tracts; 58% female). Using multilevel models, we found that approximately 82% of the variance in cognitive health is due to within-tract variation (rather than between-tract variation). Further, self-reported physical activity behavior was related to cognitive health at both the individual- and neighborhood levels, but there were no effects of the built environment (physical activity structures) or behavior-opportunity gaps on cognitive health. Likewise, there were no significant associations for substance use behavior, built environment structures, or behavior-opportunity gaps. We discuss the implications of these results for identifying prevention and intervention targets for promoting healthy cognitive aging in place.

